# Bisphenol A substitutes and obesity: a review of the epidemiology and pathophysiology

**DOI:** 10.3389/fendo.2023.1155694

**Published:** 2023-07-17

**Authors:** Shane V. Varghese, Julianne M. Hall

**Affiliations:** Frank H. Netter MD School of Medicine, Quinnipiac University, North Haven, CT, United States

**Keywords:** obesity, BPA, bps, PPARγ, adipogenesis, obesogens, endocrine disruptor obesity, obesogens keyword

## Abstract

The prevalence of obesity, a condition associated with increased health risks, has risen significantly over the past several decades. Although obesity develops from energy imbalance, its etiology involves a multitude of other factors. One of these factors are endocrine disruptors, or “obesogens”, when in reference to obesity. Bisphenol A (BPA), a known endocrine disruptor used in plastic materials, has recently been described as an environmental obesogen. Although BPA-free products are becoming more common now than in the past, concerns still remain about the obesogenic properties of the compounds that replace it, namely Bisphenol S (BPS), Bisphenol F (BPF), and Bisphenol AF (BPAF). The purpose of this review is to investigate the relationship between BPA substitutes and obesity. Literature on the relationship between BPA substitutes and obesity was identified through PubMed and Google Scholar, utilizing the search terms “BPA substitutes”, “bisphenol analogues”, “BPS”, “BPF”, “BPAF”, “obesity”, “obesogens”, “adipogenesis”, “PPARγ”, and “adipocyte differentiation”. Various population-based studies were assessed to gain a better understanding of the epidemiology, which revealed evidence that BPA substitutes may act as obesogens at the pathophysiological level. Additional studies were assessed to explore the potential mechanisms by which these compounds act as obesogens. For BPS, these mechanisms include Peroxisome proliferator-activated receptor gamma (PPARγ) activation, potentiation of high-fat diet induced weight-gain, and stimulation of adipocyte hypertrophy and adipose depot composition. For BPF and BPAF, the evidence is more inconclusive. Given the current understanding of these compounds, there is sufficient concern about exposures. Thus, further research needs to be conducted on the relationship of BPA substitutes to obesity to inform on the potential public health measures that can be implemented to minimize exposures.

## Introduction

Obesity is clinically defined as having a BMI greater than or equal to 30 kg/m2. It is a significant risk factor for the development of type 2 diabetes mellitus, coronary artery disease, hypertension, dyslipidemia, sleep apnea, stroke, osteoarthritis, and various cancers (1). Obesity also increases the risk for all-cause mortality (2). The adult population in the US has seen its obesity rates increase from 30.5% in 1999-2000 to 42.4% in 2017-2018 (3). An arguably more worrying trend can be found in the child population, which has seen rates of obesity increase from 5.2% in 1971-1974 to 18.5% in 2015-2016 (4). Worldwide, the prevalence of obesity has tripled since 1975 (5).

At its core, obesity develops from an energy imbalance caused by physical inactivity, excess dietary consumption, or both. However, the development of obesity can be influenced by a multitude of different environmental and societal factors (5). Endocrine disruptors, or “obesogens’’ when in reference to obesity, are thought to be one of the factors that play a role in the pathogenesis of obesity (6, 7). Endocrine disruptors are exogenous compounds found in the environment that can interact with endocrine pathways in the body and contribute to pathology (8). BPA, a compound used in the manufacturing of polycarbonate plastics and epoxy resins, has been shown to exhibit obesogenic activity (9). BPA-containing consumer products like metal cans, water bottles, piping, thermal paper, and dental sealants can leach BPA into the environment and food despite normal use of these products (10).

**Figure 1:**
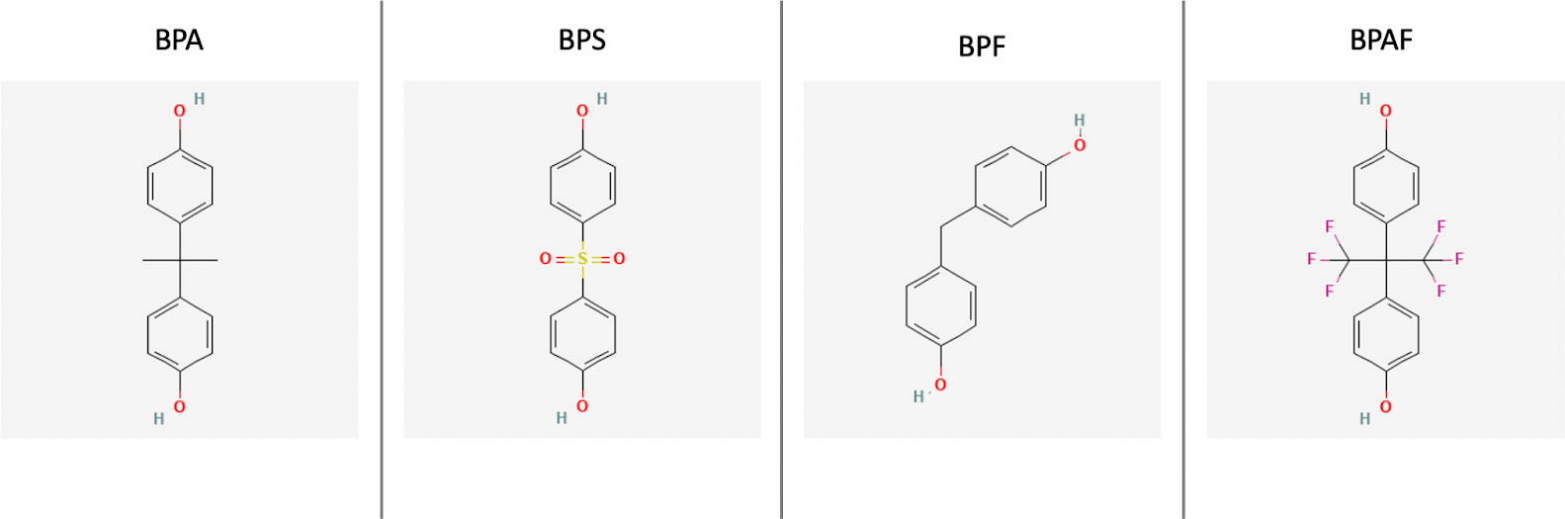
The 2D chemical structures of BPA, BPS, BPF, and BPAF (12).

While the trend of BPA-free products is more prevalent now than in the past, concern still remains about the compounds that replace it, such as Bisphenol S (BPS), Bisphenol F (BPF), and Bisphenol AF (BPAF). Both BPS and BPF have been shown to have endocrine disrupting activity with regard to their androgenicity and estrogenicity (11). The endocrine disrupting properties of these BPA substitutes are continually being investigated, including their obesogenic properties. The chemical structures of these agents are shown in **Figure 1** along with BPA (12). The purpose of this review is to assess the current literature on BPA substitutes and their relation to obesity with regards to the epidemiology of exposure and the obesogenic mechanisms of these compounds.

## Background

BPS, BPF, and BPAF have been detected in a variety of different sources of exposure. These include environmental sources such as indoor dust, water, sewage, and sediments. They have also been found in food and consumer products such as personal care items, thermal receipts papers, and recycled paper products (13). Mustard has been found to contain BPF at higher concentrations than other bisphenols (13, 14), suggesting that different foods may contain more of one type of BPA substitute than the others. Furthermore, canned foods tend to have higher concentrations of bisphenols compared to food in glass, paper, or plastic packaging (14). Given the variety of these sources of exposure, use of these products can unsurprisingly lead to higher levels of bisphenol substitutes in humans. One study found a positive association between urinary BPS concentration in adult males after fabric softener use, paint/solvent use, beef consumption, or cheese consumption within 24 hours of exposure (15).

BPA substitutes have also been detected in the human population. A 2012 study measured BPS levels of urine samples taken from populations in countries including the US, China, India, Japan, Korea, Kuwait, Malaysia, and Vietnam. Of the 315 urine samples collected, BPS was detected in 81% of them, with the highest concentrations found in residents of Japan, the US, and China (16). Another study, measuring the urinary levels of various bisphenols in residents living near a manufacturing plant in China, detected BPS, BPF, and BPAF in addition to BPA (17). Regarding the relative urinary concentrations and detection frequencies of these BPA substitutes, it appears that BPS is more prevalent than BPF. This was observed in a study that measured the levels of BPA, BPS, and BPF in 100 urine samples collected during 2009-2012 from adults in the United States. BPS had the second highest urinary concentrations and detection frequencies, after BPA, at 0.13 ng/mL and 78% respectively, whereas BPF was third at 0.08 ng/mL and 55% (18). A similar trend was seen in a separate study that looked at urine samples from the National Health and Nutrition Examination Survey (NHANES) for 2013-2014, which includes collections from both adults and children in the US. However, in their study, the median urine concentrations of BPF for children were slightly higher than BPS (19).

## Epidemiology

A few population-based studies have compared BPA substitute exposure to obesity. For the adult population ages 20 and older, Liu et al. performed a cross sectional study of 1521 adults from the NHANES for 2013-2014. They found higher BPA, BPF, and BPS concentrations in obese adults compared to nonobese adults (20). A different study used three different statistical models to assess chemical exposure and outcomes using 1269 adults from the NHANES for 2013-2014. Based on all three models, they concluded that BPA and BPS were two of three chemicals most significantly associated with obesity out of the seven chemicals studied (21).

In the adolescent and child population, Liu et al. performed a cross sectional study of 745 people ages 6-17 from the NHANES for 2013-2014. While BPF and BPA were associated with an increased prevalence of general and abdominal obesity, BPS did not have a statistically significant association with obesity (22). A different cross-sectional study included 1831 people ages 6-19 from the NHANES for 2013-2016. In this study, BPS levels were associated with an increased prevalence of general obesity and abdominal obesity. Additionally, the detection of BPF was positively correlated with an increased prevalence of abdominal obesity (23).

Other reports have examined the relationship between BPA substitutes and obesity-related diseases, like diabetes and hypertension. A case-control study from Tianjin, China compared urinary bisphenols in 251 cases of people with type 2 diabetes mellitus (T2DM) and 251 controls. They found that urinary concentrations of BPS and BPAF were positively associated with type 2 diabetes mellitus. This positive association still held true even with stratification by BMI (24). In a case-cohort study of 755 patients without T2DM at baseline and at 3 years, the detection of BPS-glucouronide, a urinary metabolite of BPS, in urine at baseline or at 3 years was associated with the development of T2DM (25). Jiang et al. focused on the relationship between bisphenol exposure and hypertension. In their study of 1437 people from Wuhan, China, they found that BPS exposure showed a positive association with hypertension (26).

As discussed above, there exists a wealth of evidence that concentrations of BPA substitutes are associated with obesity and obesity-related comorbidities. However, it should be noted that some studies have not observed this relationship. A cross-sectional study of 1521 US adults found that only BPA, but not BPF or BPS, was significantly associated with obesity at current exposure levels (20). In a population-based cohort study of 1396 pregnant women in the Netherlands, BPS and BPF were widely detected among the participants, however the investigators also observed no correlation between BPS and BPF and pre-pregnancy obesity (27). Regardless of these negative associations found in these studies, the wealth of available evidence justifies continued monitoring of these chemicals and further examination of their effects on human health.”

## Obesogenic mechanisms

While epidemiological studies are helpful in exploring the relationship between BPA substitutes and obesity on a population level, it is also important to look at studies that seek to understand how BPA substitutes are involved in the pathophysiology of obesity. Most of the available reports have focused on BPS, while a few have included BPF and BPAF as well. The underlying theme of these studies suggests that the transcription factor PPARγ, a master regulator of adipogenesis (28), is the key pathophysiological link between BPA substitutes and obesity. PPARγ is known to regulate expression of various genes that are involved in adipogenesis (29). Many of the following studies look into the obesogenic activity of BPA substitutes by focusing on their effects on PPARγ and other key adipogenic markers.

### Evidence for obesogenic activity of BPA substitutes via PPARγ

**Figure 2:**
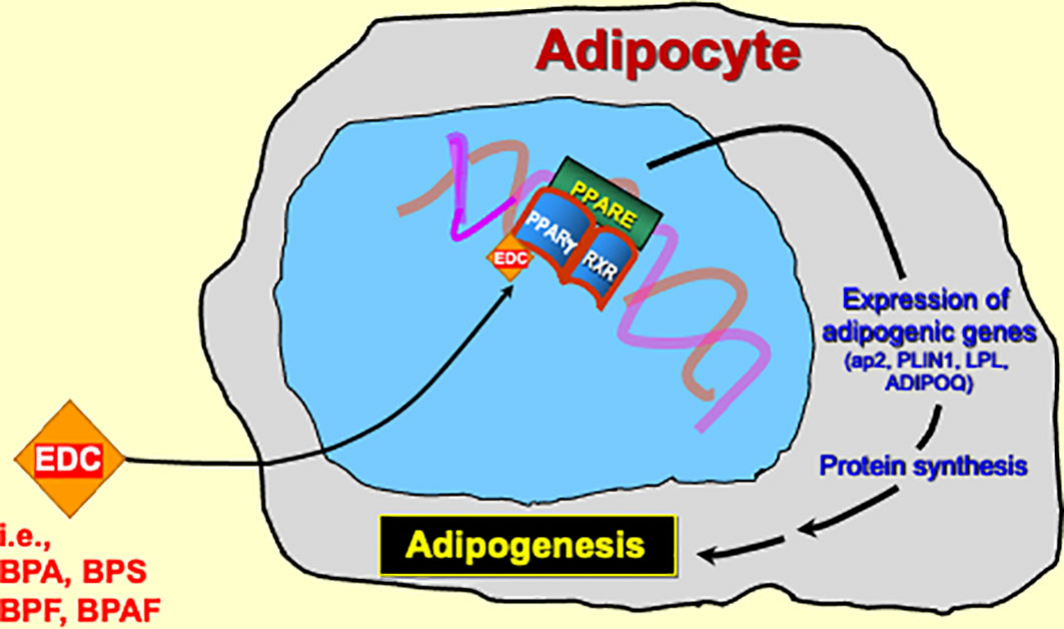
The pathophysiological link between BPA substitutes and obesity is most likely through PPARγ activation.

Several reports have used established models of adipogenesis to delineate the mechanism by which BPS exerts obesogenic activity via PPARγ (**Figure 2**). Initial reports revealed a potential link between BPS, PPARγ, and adipogenesis. One such study found that exposing human primary subcutaneous preadipocytes to various BPS concentrations led to lipid accumulation and increased mRNA and protein levels of various adipogenic markers, including Adipocyte protein 2 (aP2), Sterol regulatory element binding protein 1 (SREBP-1), Lipoprotein lipase (LPL), and Perilipin 1 (PLIN1). When investigating a mechanistic explanation, they found that BPS could activate PPARγ transcriptional activity when assessed on a PPARγ-response element (PPRE) reporter plasmid. BPS was also observed to increase human aP2 promoter activity, in the presence of both PPARγ and the glucocorticoid receptor. One potential limitation to their findings is that they only observed statistically significant effects at concentrations higher than what humans are normally exposed to (30).

An additional study in 3T3-L1 adipocytes reported that BPS, but not BPA, caused upregulation of PPARy-coactivator-1alpha and increased lipid content. The effects were manifest at in the pico- and nanomolar ranges which are consistent with those levels of BPS detected in human blood (31). In a subsequent report, *in vitro* observations were corroborated in mice, where BPS exposure promoted lipid accumulation and weight increase, exacerbating high-fat-diet-induced obesity (32).

Further deleterious effects of BPS were recently uncovered in a study using computational systems biology modeling of scientific literature. The authors reported that 48 adverse pathways were associated with BPS exposure, including outcomes encompassing endocrine and reproductive health, metabolic dysregulation, and obesity. Analysis of the ToxCast database revealed positive associations between BPS and PPARγ activity, and the authors proposed that BPS-activation of PPARγ as a mechanism for the observed relationship of BPS exposure with increased adipogenesis and obesity (33).

A different study found that exposing murine 3T3-L1 preadipocytes to BPS induced greater lipid accumulation and mRNA and protein expression of adipogenic markers compared to BPA, suggesting that BPS is a more potent obesogen. Using a PPRE dependent luciferase construct in COS-7 cells, they also found that both BPS and BPA activated PPARγ by about 1.5-fold. In addition, competitive inhibition of rosiglitazone activated PPARγ could be achieved with BPS (32), but not BPA, suggesting that there might be a difference in how they each interact with PPARγ. Finally, the ability of the selective PPARγ antagonist GW9662 to inhibit BPS, BPA, and rosiglitazone induced adipogenesis, but not dexamethasone induced adipogenesis, suggests that PPARγ is an integral component of both BPS and BPA’s obesogenic mechanisms (34).

Gao et al. found that human macrophages (THP-1 cells) and human embryonic kidney (HEK293T) cells exposed to BPS showed increased activity and expression of PPARγ and its downstream target genes. BPS also induced increased expression of PPARγ target genes in mouse liver tissue and altered lipid metabolism *in vivo (* 35). Wang et al. used a human embryonic-derived mesenchymal stem cell model to test the effects of exposure to potential obesogens like BPS. BPS exposure led to intracellular triglyceride accumulation and increased expression of adipogenic genes such as PLIN1, PPARγ, and ap2 (36).

Although this review focuses on PPARy, in the past 3 decades, there is increasing evidence for the estrogen receptors (ERs) as key players in the regulation of metabolism and energy utilization (37). Indeed, the decline in estrogens that occur in menopause is associated with the loss of subcutaneous fat and an increase in abdominal fat (38). Furthermore, estrogen receptor knockout mice display increased adiposity, elevated cholesterol, insulin resistance, and glucose intolerance (39, 40).

BPA is an established ER ligand, and many of the endocrine disrupting effects of BPA are attributed to its estrogenic activities. With regards to adipogenesis, BPA has been observed to increase differentiation of human adipocyte stem cells into mature adipocytes in a manner requiring ER activation (41).

While BPA is a weak agonist of the nuclear ERs, BPA is a potent activator of rapid, nongenomic estrogenic signaling mediated through membrane ERs (42, 43). In a recent report, BPS and BPF were shown to rapidly increase insulin release from murine pancreatic β-cells, and after prolonged stimulation, alter expression of membranous ion channel subunits. Both of these activities required ERs, suggesting that both membrane and nuclear ERs may be involved in the endocrine disrupting effects of BPA and its substitutes (44).

### Evidence for obesogenic effects of BPA substitutes as a consequence of prenatal exposure

Another mechanism by which BPS might display obesogenic activity is through potentiation of high-fat diet weight gain as a consequence of prenatal exposure. In one study, high-fat fed male mice with prenatal exposure to BPS showed increased gonadal white adipose tissue hypertrophy and expression of adipogenic gene like PPARγ, CCAAT enhancer-binding protein alpha (CEBPA), ap2, LPL, and Adiponectin (ADIPOQ) compared to mice fed with a standard diet (45). Likewise, a different study produced results that similarly suggested that prenatal exposure to BPS predisposes male mice offspring to high-fat diet induced weight gain. However, in this study, BPS exposure led to a slight decrease in the mRNA expression of PPARγ and hormone sensitive lipase (32).

### Evidence for BPA substitute effects on adipose tissue deposition

BPS exposure may also lead to adipose depot-dependent effects. Peshdary et al. found that exposing human primary subcutaneous and omental preadipocytes to BPS led to an increase in the mRNA levels of adipogenic markers such as ap2, LPL, SREBP-1, and PLIN. They also found that the effect of BPS on these adipogenic markers differed from the thiazolidinedione Rosiglitazone (a PPARγ agonist) with regard to the specific adipose tissue depot involved, subcutaneous vs. omental or visceral (46). This suggests that BPS may have distinct depot-dependent effects on adipogenesis that differ from thiazolidinediones, which have been shown to increase subcutaneous fat instead of visceral fat (47–49). Other studies show how BPS exposure leads to increased gonadal white adipose tissue and epididymal white adipose tissue hypertrophy, both of which are also types of visceral fat (45, 50). Given the well-established relationship between visceral obesity and metabolic disease (51), this points to a unique relationship between BPS and obesity that is different from other PPARγ agonists such as those used medically.

Compared to BPS, the obesogenicity of BPF is more unclear. One study compared the effects of BPA, BPS, and BPF on murine 3T3-L1 preadipocyte differentiation and followed up with an *in vivo* study of male mice administered 3 different dosages of BPF for 12 weeks. BPS had the biggest effect on lipid accumulation and preadipocyte differentiation, increasing the expression of different adipogenic markers including PPARγ. BPA followed BPS in these effects. In contrast, BPF had no effect on lipid accumulation and instead, decreased expression of several adipogenic markers. BPF also induced lower weight gain in male mice compared to controls (52). On the other hand, other studies seem to suggest that chlorinated BPS or BPF function as PPARγ agonists. Notably, the chlorinated byproducts of BPS and BPF were found to increase PPARγ activity more than the parent compounds. This increase in PPARγ activity corresponded directly to the degree of BPS or BPF chlorine substitution, i.e., tetra-chloro-BPS > tri-chloro-BPS (53, 54). Interestingly, both BPS and BPF seem to share this characteristic with BPA, which has also been found to display greater PPARγ activity when the compound is more halogenated (55, 56). Given that the chlorination of water containing BPA can result in the formation of its chlorinated byproducts (57), these findings warrant concern about whether the same is true for BPA substitutes and what the downstream endocrine disrupting effects may occur.

BPAF, like BPF, has unclear obesogenicity given the relatively few studies. One study observed that BPAF had some partial agonist activity on PPARγ, but also increased IFNγ activation of STAT1. From this, they suggest that BPAF has pro-inflammatory effects that diminish mitochondrial activity in human adipocytes (58). A different report found that BPAF and its glucuronidation product BPAF-glucuronide (BPAF-G) lead to lipid accumulation and increased expression of the adipogenic markers adiponectin, ap2, and CEBPA, but not PPARγ, in murine 3T3L1 preadipocytes. In this study, BPAF also lacked PPARγ agonist activity, while BPAF-G displayed PPARγ antagonist activity (59). Regardless, if BPAF does indeed display obesogenic activity, available evidence supports a possible pro-inflammatory mechanism.

## Conclusion

**Figure 3:**
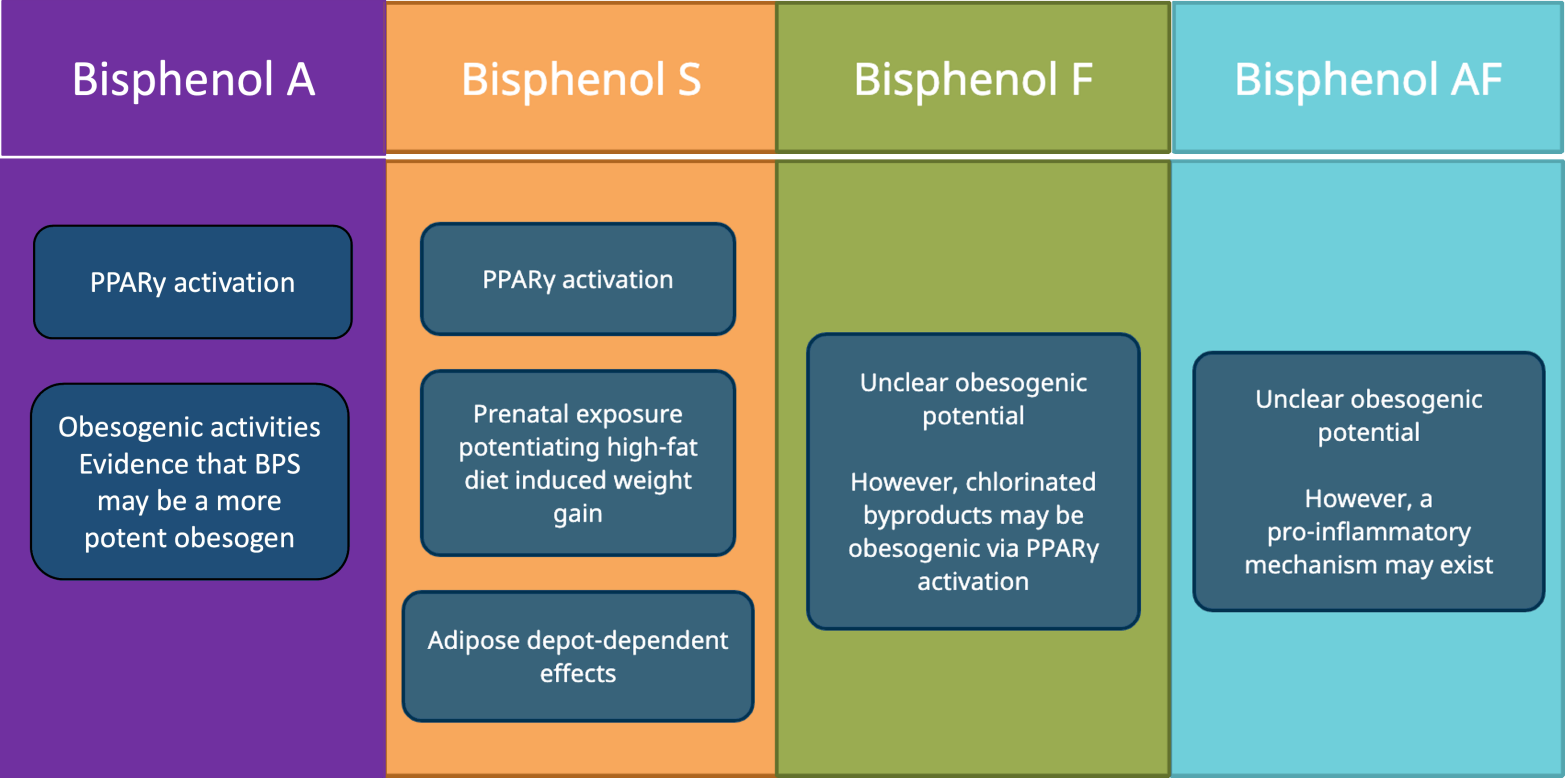
Potential mechanisms by which BPA substitutes act as obesogens.

With the increasing prevalence of BPA-substitutes in environmental sources, food, and consumer products, more research needs to be performed to study their endocrine-disrupting effects. Of these BPA substitutes in distribution, BPS is most prevalent in the human population. Currently, there are only a few epidemiological studies on the relationship between BPA substitutes and obesity. While some of these studies have established a positive relationship between BPA substitutes and obesity or obesity-related disease, others have been mixed. There are relatively more studies exploring the pathophysiological relationship between BPA substitutes and obesity, most of which concern BPS (**Figure 3**). A few ways BPS might be involved in the pathogenesis of obesity include PPARγ activation, prenatal exposure that potentiates high-fat diet induced weight-gain, and adipose depot- dependent effects. For BPF and BPAF, the evidence is relatively sparse on whether there is a pathophysiological link between these BPA substitutes and obesity.

Given what is currently known about these BPA substitutes, a question that remains is whether this warrants public health measures to regulate exposure to these exogenous compounds. Additionally, how should these public health measures be implemented? In June of 2020, Switzerland became the first country to ban BPS in addition to BPA in thermal paper (60). While the question of how-to best address BPA substitutes is continually debated, it will be increasingly important to further advance our understanding of their endocrine disrupting effects, especially with respect to obesogenicity. There is currently a paucity of epidemiological studies on these substitutes. Further research on BPF and BPAF will also need to be performed if they become more prevalent in the population.

## Author contributions

SV played a primary role in defining the project, researching the literature, developing Figures, and drafting the manuscript. JH played an advisory role in guiding developing of the project, outline, and a primary role in writing and editing the manuscript, developing figures, and addressing reviewer comments. All authors contributed to the article and approved the submitted version.
